# Regulation of CTLA-4 recycling by LRBA and Rab11

**DOI:** 10.1111/imm.13343

**Published:** 2021-06-06

**Authors:** Daniel Janman, Claudia Hinze, Alan Kennedy, Neil Halliday, Erin Waters, Cayman Williams, Behzad Rowshanravan, Tie Zheng Hou, Shane Minogue, Omar S Qureshi, David M. Sansom

**Affiliations:** 1 institute of Immunity and Transplantation, University College London, London, United Kingdom; 2 Institute of Liver and Digestive Health, University College London, London, United Kingdom; 3 Celentyx Ltd, Birmingham, United Kingdom

**Keywords:** CTLA-4, LRBA, Rab GTPase, Rab11, trafficking, recycling, T cells

## Abstract

CTLA-4 is an essential regulator of T cell immune responses whose intracellular trafficking is a hallmark of its expression. Defects in CTLA-4 trafficking due to LRBA deficiency cause profound autoimmunity in humans. CTLA-4 rapidly internalises via a clathrin-dependent pathway followed by poorly characterised recycling and degradation fates. Here we explore the impact of manipulating Rab GTPases and LRBA on CTLA-4 expression to determine how these proteins affect CTLA-4 trafficking. We observe that CTLA-4 is distributed across several compartments marked by Rab5, Rab7 and Rab11 in both Hela and Jurkat cells. Dominant negative (DN) inhibition of Rab5 resulted in increased surface CTLA-4 expression and reduced internalisation and degradation. We also observed that constitutively active (CA) Rab11 increased, whereas DN Rab11 decreased, CTLA-4 surface expression via an impact on CTLA-4 recycling, indicating CTLA-4 shares similarities with other recycling receptors such as EGFR. Additionally, we studied the impact of manipulating both LRBA and Rab11 CTLA-4 trafficking. In Jurkat cells LRBA deficiency was associated with markedly impaired CTLA-4 recycling and increased degradation that could not be corrected by expressing CA Rab11. Moreover LRBA deficiency reduced CTLA-4 co-localisation with Rab11 suggesting that LRBA is upstream of Rab11. These results show that LRBA is required for effective CTLA-4 recycling by delivering CTLA-4 to Rab11 recycling compartments and in its absence CTLA- 4 fails to recycle and undergoes degradation.

## 2 Introduction

CTLA-4 is an essential regulatory protein involved in control of T cell immune responses and prevention of autoimmunity. CTLA-4 is expressed on T lymphocytes, and in particular on a subset of cells termed regulatory T cells (Treg) (1). The loss of Treg (2) or the loss of CTLA-4 specifically from Treg in mice (3) triggers fatal autoimmunity. Similarly, heterozygous missense mutations in CTLA-4 or mutations in the trafficking protein LPS-responsive beige-like anchor protein (LRBA) (which affects CTLA-4 expression) both cause clinically significant autoimmunity (4–6). In addition CTLA-4 is a key target for cancer immunotherapy, whereby antibodies to CTLA-4 revolutionised cancer treatment by triggering (auto)-immune responses to tumours (7). Understanding the cell biology of CTLA-4 is therefore of considerable medical significance.

CTLA-4 binds to two transmembrane ligands (CD80 and CD86), which are expressed on the cell surface of antigen presenting cells including dendritic cells and B cells. The two ligands have distinct avidities for CTLA-4 with CD80 being a high affinity ligand and CD86 being low affinity (8–10).

Despite binding to cell surface ligands, CTLA-4 has a pattern of expression that is unusual for immune receptors, being largely intracellular as a result of clathrin-mediated endocytosis (11–14). The purpose of this endocytic control may relate to the observation that CTLA-4 has the ability to carry out transendocytosis of its ligands (15). Here, transmembrane ligands are removed from APCs and transferred to CTLA-4 expressing T cells for destruction in lysosomes. This ligand depleting mechanism has the capacity to supress immune responses by preventing a second, activating receptor, CD28, from binding to CD80 and CD86, which it shares with CTLA-4. The importance of CTLA-4 trafficking to its function has been recently highlighted in individuals defective in a protein, LRBA, who develop early onset autoimmunity (6). This results from a deficiency in CTLA-4 and defective Treg function, caused by excessive degradation of CTLA-4. Thus correct trafficking of CTLA-4 appears critical in order to sustain its function in controlling ligand expression.

Studying the trafficking of archetypal endocytic receptors such as EGFR has revealed that the intracellular itinerary and sorting steps between cellular compartments are controlled by a family of Rab GTPases (16). Accordingly, different cellular compartments are marked by Rab GTPase expression, for example Rab5 marks early endosomes and Rab11 marks recycling endosomes etc. Thus different Rab markers can be useful in identifying the pathways used by endocytic receptors. In addition, manipulating Rab activity by overexpressing active or dominant inhibitory Rab GTPases can be a useful approach to studying the impact of these pathways on proteins of interest (17–21). CTLA- 4 has a dynamic intracellular sorting itinerary involving internalisation via a canonical YxxM AP-2 binding motif and recycling or degradation via steps that are not well defined (12,22). Interestingly whilst intracellular trafficking is a highly conserved feature in mammalian CTLA-4, this appears to have evolved from a non-endocytic form seen in fish (23).

To better understand the pathways that are important to CTLA-4 trafficking we have studied its localisation and response to Rab GTPase manipulation in HeLa cells and Jurkat cell lines. The epithelial cell morphology of HeLa cells enables more robust co-localisation studies due to extensive cytoplasm in comparison to lymphocytes, whereas the T lymphocyte lineage of Jurkat cells enables validation of concepts seen in adherent cells in a more physiologically relevant cell type. The present studies reveal broad concordance between the observations made in the two cell types that align with previous studies in CHO cells (12), indicating that CTLA-4 trafficking behaviour is well conserved across cell types. Accordingly, CTLA-4 is rapidly endocytosed with most of the protein being intracellular at steady state. Inhibition of Rab5 caused an increase of CTLA-4 at the cell surface and reduced the overall degradation of CTLA-4. Similarly, inhibition of Rab7 reduced the degradation of CTLA-4 in favour of its recycling. We also observed that CTLA-4 was recycled in a manner dependent on Rab11 and that this required the participation of LRBA. Accordingly, deletion of LRBA reduced the entry of CTLA-4 into Rab11 compartments. Overall we find that in the absence of ligand, constitutive CTLA-4 trafficking adopts many of the hallmark pathways seen for other clathrin-mediated endocytic receptors. However, CTLA-4 is specifically dependent on LRBA to permit effective recycling via a Rab11 compartment.

## 3 Materials and Methods

### Cell Culture

HeLa cells were grown in DMEM (Invitrogen) supplemented with 10% FBS (Sigma), 2 mM L- glutamine (Sigma), and 100U/ml penicillin and streptomycin (Sigma), in a humidified 37°C/5% CO_2_ environment, and passaged by trypsinisation. Jurkat cells were grown in RPMI 1640 (Invitrogen) supplemented with 10% FBS, and 100U/ml penicillin and streptomycin, in a humidified 37°C/5% CO2 environment, and passaged by aspirating excess cells.

### Generation of CTLA-4+ cell lines

HeLa and Jurkat cells were transduced with CTLA-4 using virus produced using Phoenix-A cells. In short, retrovirus containing supernatant was collected from Phoenix-A packaging cells transfected with MP71 CTLA-4 and VSV-G envelope vectors using FUGENE6 (Roche Molecular Biochemical). This virus was then used to spinoculate HeLa and Jurkat cells in non-tissue culture treated 6-well plates coated with RetroNectin (TaKaRa). Successful transduction was tested by flow cytometry and clones or populations with the desired CTLA-4 expression level were obtained by single cell cloning or FACS sorting.

### Rab-GFP overexpression

HeLa or Jurkat cells were plated in 24-well plates in antibiotic-free media and transfected with 0.8 μg Rab-GFP construct DNA using Lipofectamine 2000 (Invitrogen) according to the manufacturer’s instructions. The cells were then incubated at 37°C for 24hrs to allow expression of the Rab construct before being used for experiments.

The following Rab-GFP constructs were acquired from Addgene and generated by Marci Scidmore (Rzomp et al; Cortes et al) (Rab4 WT, DN, CA; Rab5 WT; Rab11 CA), Sergio Grinstein (Bohdanowicz et al) (Rab5 DN, CA), and Richard Pagano (Choudhury et al) (Rab9 WT, DN; Rab11 WT, DN): Rab4 WT (Addgene plasmid #49434), Rab4 DN (#49476), Rab4 CA (#49475), Rab5 WT (#49888), Rab5 DN (#35141), Rab5 CA (#35140), Rab9 WT (#12663), Rab9 DN (#12664), Rab11 WT (# 12674), Rab11 DN (#12678), Rab11 CA (#49553). Rab7-GFP constructs were kindly provided by Angela Wandinger-Ness (University of New Mexico, USA).

### LRBA siRNA knockdown

HeLa cells were plated in a 24-well plate and transfected with 20 nM siRNA, ON-TARGETplus Human LRBA SMARTpool (L-012751-00) or ON-TARGETplus Non-targeting SMARTpool (D- 001810-10-05) (Dharmacon GE Life Sciences), using jetPRIME (Polyplus-transfection) according to the manufacturer’s instructions. The cells were then incubated at 37°C for 48hrs, with the media replaced after 24hrs, before being trypsinised and used for experiments.

### LRBA CRISPR knockout

CRISPR-Cas9 targeting was used for the generation of LRBA-deficient CTLA-4 Jurkat lines. CRISPR-Cas9 target sites were designed using CHOPCHOP (https://chopchop.rc.fas.harvard.edu), and *in vitro* sgRNA syntheses containing the relevant target site were performed using the EnGen sgRNA Synthesis Kit, *S. pyogenes* (NEB) according to the manufacturer’s instructions. Transcribed sgRNAs were purified using the RNA Clean & Concentrator kit (Zymo Research) following the manufacturer’s instructions.

For generation of cell lines, 500 ng sgRNA and 2 μg Cas9 protein (TrueCut™ Cas9 Protein v2) were electroporated into 2x10^5^ target cells using the Neon™ Transfection System (Thermo Fisher Scientific) under the following conditions: voltage (1600 V), width (10 ms), pulses (three), 10 μl tip, and Buffer R. Cells were allowed to recover for 3-5 days prior to screening for KO by flow cytometry. This approach generally yielded heterozygous and homozygous KO of the target gene in 70-95% of the cells.

### Flow cytometry

All experiments were analysed on a BD LSR Fortessa (BD Bioscience, New Jersey, USA) and then processed using FlowJo Version 10 (TreeStar).

### CTLA-4 Surface:Total staining

Cells were incubated on ice with anti-CTLA4-PE (BNI3, BD Biosciences) to stain surface CTLA-4. The cells were then fixed with 3% paraformaldehyde in PBS and permeabilised with 0.1% saponin in media before incubating in 0.1% saponin in media with (total) or without (surface) anti-CTLA4-PE for 20mins.

### CTLA-4 degradation with antibody feed

Cells were incubated at 37°C with anti-CTLA-4 PE in media for 1hr. Cells were then washed and incubated at 37°C for up to 4hrs, with or without 10nM BafA1 (Sigma). Cells were fixed with 3% PFA in PBS and permeabilised with 0.1% saponin in media before being washed and analysed.

### CTLA-4 degradation with CHX

Cells were treated with 50 μg/ml cycloheximide (CHX) in media for up to 4hrs and fixed with 3% PFA in PBS. The cells were then permeabilised with 0.1% saponin in media and stained with anti- CTLA-4 PE in media with 0.1% saponin for 20mins. The cells were then washed and analysed.

### CTLA-4 Surface and Recycling assay

Cells were incubated at 37°C with unlabelled anti-CTLA-4 (Ticilimumab, a gift from Pfizer) in media for 1hr to tag surface and internalised CTLA-4, and then washed with ice cold media and placed on ice. Antibody-bound surface CTLA-4 was stained with Goat anti-Human IgG-Alexa Fluor 647 (Thermo) in media on ice for 10mins in the dark. Cells were either washed and analysed for surface stain to provide a baseline or transferred to 37°C to allow trafficking to resume in the presence of Goat anti-Human IgG-Alexa Fluor 647. Recycling of antibody-bound CTLA-4 was detected as the increase in Alexa Fluor 647 at the time points shown. In LRBA KO Jurkat cells, after the 37°C recycling stain, cells were fixed with 3% paraformaldehyde in PBS and permeabilised with 0.1% saponin in media. The cells were then stained for total CTLA-4 (F-8 PE, Santa Cruz) and with rabbit anti-LRBA for 30mins, followed by Donkey anti-Rabbit IgG-Alexa Fluor 488.

### LRBA patient CD4+ T cell staining

Peripheral blood mononuclear cells (PBMCs) were isolated from whole blood using Ficoll-Paque PLUS (GE healthcare, Buckingham, UK) density gradient centrifugation. CD4+ T cells were purified using EasySep® Human CD4+ T cell enrichment kit (StemCell Technologies, Meylan, France) according to manufacturer’s instructions. Cells were washed with FACS buffer (2% FBS in PBS) and then stained with anti-CD4 AlexaFluor 700 (RPA-T4, BD biosciences), anti-CD45RA PerCP Cy5.5 (HI100, eBioscience, California, USA) and anti-CTLA4-PE (BNI3, BD) in FACS buffer at 4°C for 30mins in the dark. Cells were washed and stained using anti-FoxP3-APC (236A/E7, eBioscience) and the eBioscience FoxP3 staining kit according to manufacturer’s instructions.

### Confocal Microscopy

Images were taken on a Nikon Eclipse Ti confocal inverted laser scanning microscope equipped with a ×60 oil-immersion objective with excitation at 405nm, 488nm and 561nm. Constant laser powers and acquisition parameters were maintained throughout individual experiments for analysis. All images were processed using ImageJ (Wayne Rasband, NIH, USA) and analysed using CellProfiler (Broad Institute, USA). For quantification, pipelines were developed to automatically mask cells and vesicles, and then to calculate the number of vesicles in one channel that colocalised with vesicles in another. All confocal images shown are representative of at least 6 micrographs taken from at least 3 independent experiments, with at least 70 cells being analysed per condition.

### Cell setup

Cells were imaged in glass-bottomed 96-well SensoPlate microplates (Greiner Bio-one Ltd). When staining HeLa cells, 1×10^4^ cells were added per well and incubated for 2 days. When staining Jurkat cells, 1.5×10^5^ cells/well were incubated on ice in 3.7% formaldehyde 20 mM HEPES in PBS for 10mins, added to poly-L-lysine coated wells and centrifuged (350g, 5min, 4°C) to make them adhere to the glass-bottomed wells.

### Staining

Cells were fixed with 3.7% formaldehyde 20 mM HEPES in PBS for 10min. This step was skipped when working with Jurkat cells as they were fixed during the setup of the cells. The formaldehyde was quenched with 10 mM Tris in PBS for 10mins, and the cells were then permeabilised for 5mins with PBS containing 0.05% Tx-100. The cells were then blocked with 3% BSA in PBS for 20mins and then stained with primary antibodies (Ticilimumab, anti-LRBA (Atlas Antibodies), anti-Rab5, anti-Rab7, anti-Rab9 (all Cell Signaling Technology), anti-Rab11 (Invitrogen)) in 3mg/ml BSA in PBS for 1hr at RT. Next, goat anti-human IgG-AlexaFluor 546, donkey anti-rabbit IgG-AlexaFluor 488 (both Invitrogen), Hoechst 33342 (Thermo Fisher Scientific) and 2.5 μM CellTrace Violet (Molecular Probes) in 3mg/ml BSA in PBS were added to the cells for 45mins. All steps up to and including the 3% BSA block were carried out on ice, all the following steps were at RT, and the cells were washed 3 times with PBS between each step.

### Graphing and Statistical Analysis

Graphs were generated and statistical analysis was performed using GraphPad Prism (GraphPad Software, Inc., CA, USA), using unpaired T-tests, or one-way or two-way ANOVA with appropriate multiple comparisons tests.

## 4 Results

### HeLa and Jurkat cells have similar intracellular CTLA-4 distributions

**Figure 1:**
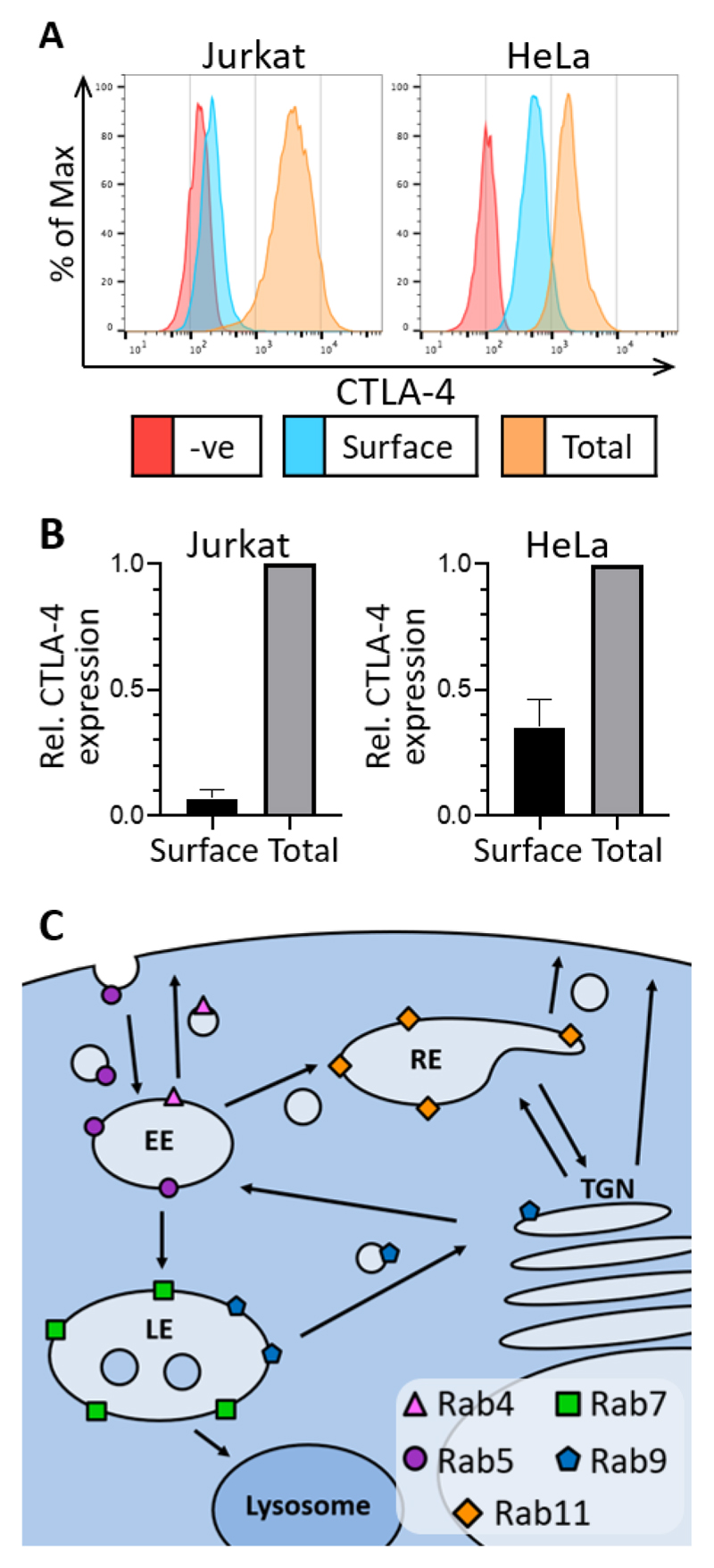
CTLA-4 is mainly intracellular in HeLa and Jurkat Cells **A**: CTLA-4 expressing Jurkat and HeLa cells, or untransduced negative controls, were stained for surface or total CTLA-4 and analysed by flow cytometry. **B**: Graphs showing quantification of the relative surface or total CTLA-4 staining in Jurkat and HeLa cells. Data shown as mean ± SD, n=3. **C**: Diagram showing the intracellular localisation of different Rab GTPases. EE - early endosome, LE - late endosome, RE - recycling endosome, TGN - trans Golgi network.

In order to interact with its ligands on antigen-presenting cells CTLA-4 needs to be present at the plasma membrane yet it displays a mainly intracellular distribution in most cell types (11,12,15,24,25). To understand control of CTLA-4 localisation in more detail we stably transduced both Jurkat and HeLa cells with CTLA-4 and stained them for surface or total CTLA-4 and analysed by flow cytometry (Fig. 1A). As previously seen with primary T cells, the majority of CTLA-4 is intracellular in both lines, with only around 10% expressed on the cell surface in Jurkat cells (Fig. 1B). In HeLa cells the fraction of CTLA-4 resident at the plasma membrane was somewhat higher (35%). Therefore whilst the overall endocytic nature of CTLA-4 is preserved in both cell types the surface to total ratios differ slightly, likely depending on the relative rates of exocytosis, endocytosis and of traffic through recycling compartments. We therefore pursued analysis in both cell types to provide a comparison of CTLA-4 behaviour and its colocalisation with markers of intracellular compartments defined by Rab GTPase expression (Fig. 1C).

### CTLA-4 colocalises with multiple Rab GTPases across intracellular compartments

**Figure 2:**
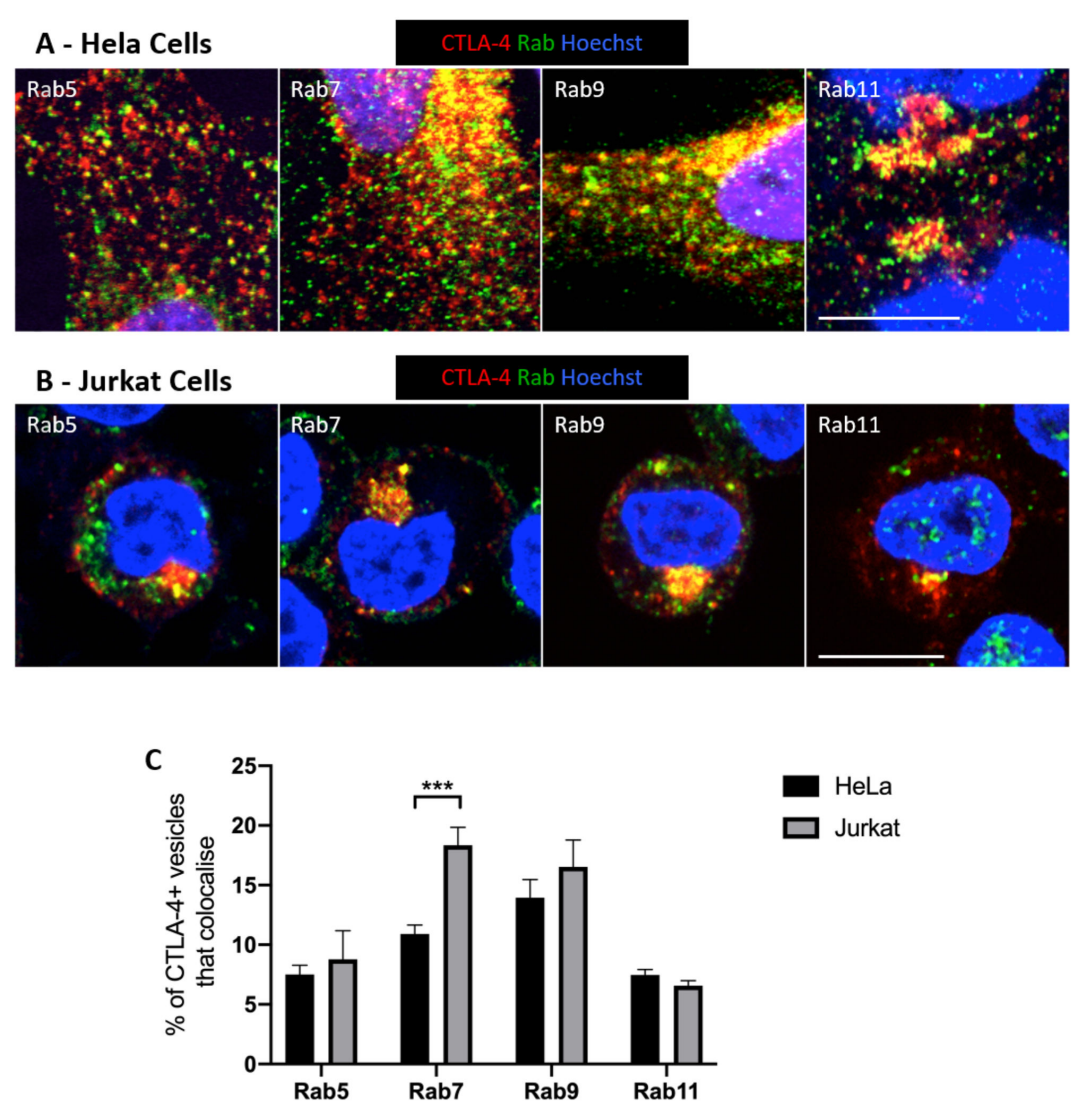
CTLA-4 colocalises with endosomal Rab GTPases **A,B:** CTLA-4 transduced HeLa (A) or Jurkat (B) cells were fixed, permeabilised and stained with human anti-CTLA-4 Ab and rabbit anti-Rab5, anti-Rab7, anti-Rab9 or anti-Rab11 Abs followed by goat anti-human IgG-AlexaFluor546, donkey anti-rabbit IgG-AlexaFluor488, Hoechst and CTV. Cells were analysed by confocal microscopy. Scale bars = 10μm. **C**: Graph showing quantification of the colocalisation of CTLA-4 vesicles with Rab vesicles in (A) or (B). Data shown as mean ± SEM, n=3. *** p<0.005 determined by two-way ANOVA with Sidak’s multiple comparison test.

To characterise CTLA-4 localisation in more detail, cells were stained for CTLA-4 and a panel of endogenous Rab GTPases and imaged by confocal microscopy (Fig. 2A and B). CTLA-4 showed substantial colocalisation with Rab GTPases in both cell types indicating it is distributed across many cellular compartments. The lack of colocalisation seen between CTLA-4 and the autophagy protein LC3 indicated that the colocalisation of CTLA-4 with the Rabs was specific (Fig. S1). Quantification of the colocalisation using CellProfiler revealed that CTLA-4 localisation was similar in HeLa and Jurkat cells, suggesting that despite differences in surface-total ratio, the trafficking pathways adopted by CTLA-4 were similar between cell types but with slightly different steady state distributions (Fig. 2C). Notably, the colocalisation of CTLA-4 with Rab7 appeared to be higher in Jurkat cells than HeLa cells (18% vs 10%). This suggests that CTLA-4 trafficking may be biased towards late endosomes en-route to lysosomal degradation in Jurkat cells, consistent with its lower steady state plasma membrane expression. Colocalisation with Rab9 also indicated that CTLA-4 traffics via late endosomes whereas colocalisation of CTLA-4 with both Rab5 and Rab11 also indicated its presence in early (Rab5) and recycling (Rab11) endosomes. This suggests that CTLA-4 may recycle via the classical ‘slow’ recycling (Rab11+) route as used by the canonical recycling transferrin receptor (26).

### Rab5 and Rab11 regulate CTLA-4 surface expression

**Figure 3:**
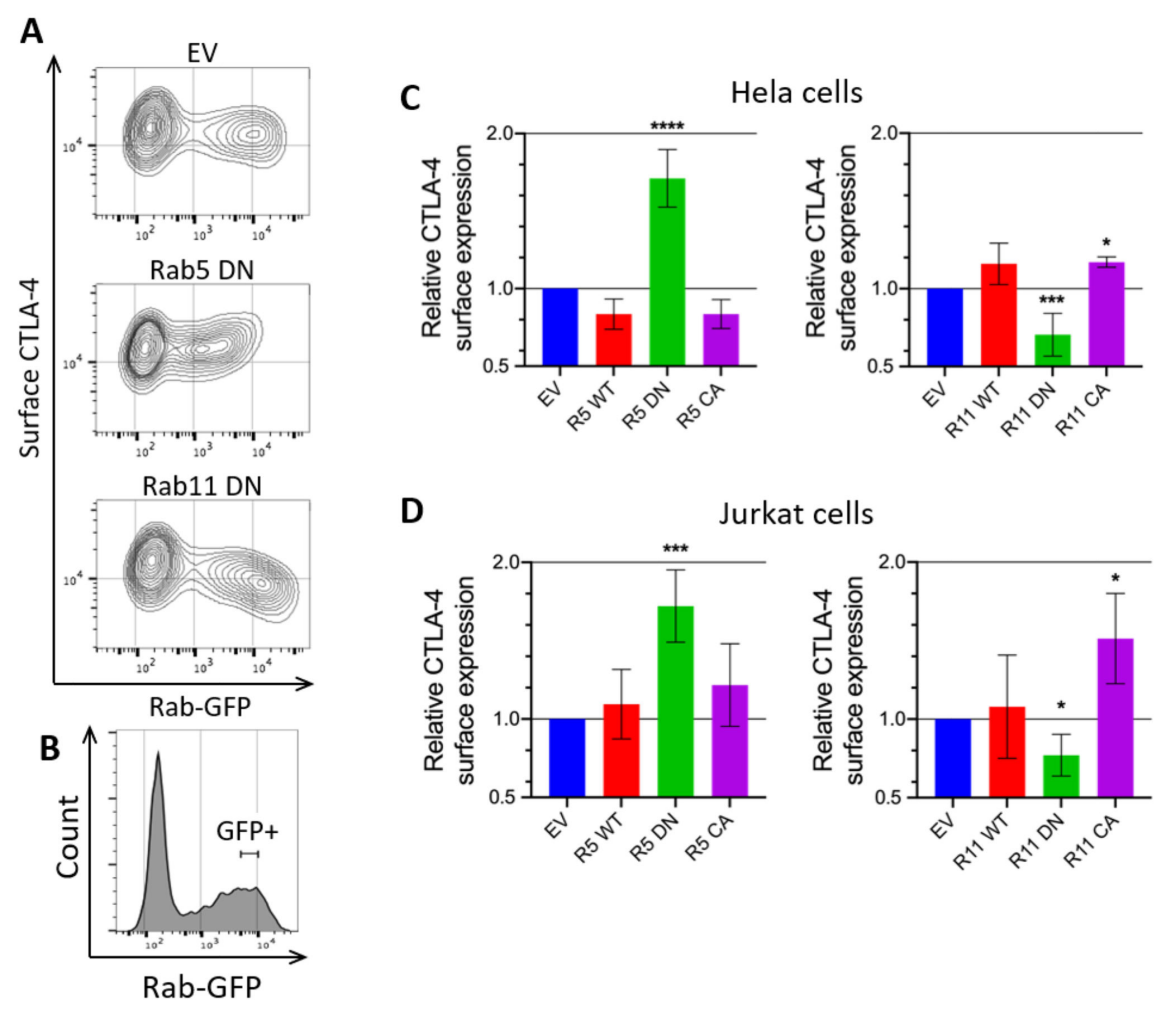
CTLA-4 surface expression is regulated by Rab5 and Rab11 CTLA-4 transduced HeLa or Jurkat cells were transfected with Rab-GFP constructs (empty vector - EV; wild type - WT; dominant negative - DN; or constitutively active - CA) for 24hrs, and then stained for surface CTLA-4 expression. **A**: Flow cytometry contour plots of HeLa cells showing Rab- GFP vs surface CTLA-4 expression. **B**: Flow cytometry plot showing the GFP+ gate used. **C,D:** Graphs showing surface CTLA-4 expression relative to empty vector control in GFP+ gated HeLa (C) or Jurkat (D) cells. Data shown as mean ± SD, C: n ≥ 3, D: n ≥ 4. Differences between conditions and EV determined by one-way ANOVA and Dunnett’s multiple comparisons test; * p<0.05, ***p<0.005, ****p<0.001.

To investigate the functional involvement of the various Rab GTPases that colocalised with CTLA-4 we adopted the approach of transiently overexpressing wild type (WT), dominant negative (DN) or constitutively active (CA) GFP-tagged Rab GTPase constructs, and measured surface CTLA-4 against GFP expression (Fig. 3A). Quantification of surface CTLA-4 MFI gated on a population expressing a tightly defined level of GFP (comparable between experiments) (Fig. 3B) showed that CTLA-4 surface expression was up-regulated by Rab5 DN and down-regulated by Rab11 DN in HeLa cells (Fig. 3C), a finding recapitulated in Jurkat cells (Fig. 3D). Other Rab constructs tested did not have a significant impact (Fig. S2). The subtle differences in Rab5 and Rab11 effects between cell types may suggest some differences in the relative efficiency of these pathways, however the overall trends were consistent. Taken together we concluded that Rab5 DN inhibited endocytosis, resulting in an increase in CTLA-4 surface expression whereas the opposite impacts of Rab11 DN and Rab11 CA in decreasing and increasing surface CTLA-4 showed that CTLA-4 recycles to the cell surface via a Rab11 regulated pathway.

### Rab5 and Rab7 regulate CTLA-4 degradation

**Figure 4:**
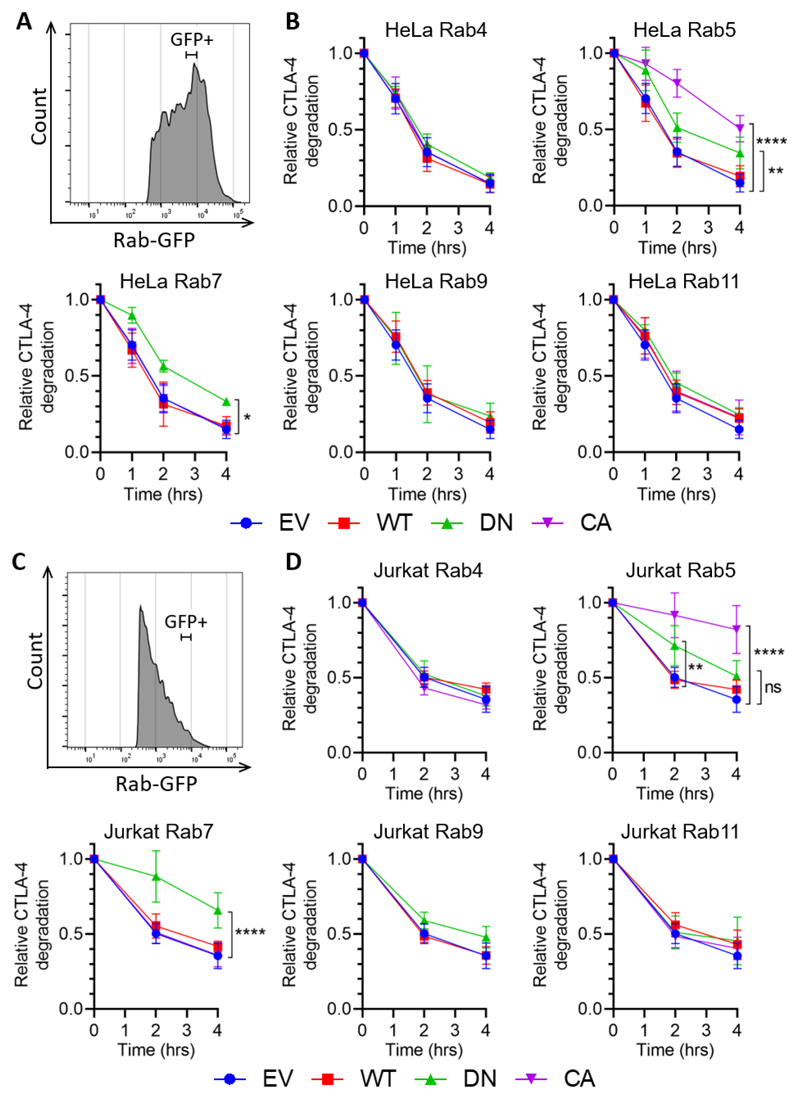
CTLA-4 degradation is regulated by Rab5 and Rab7 CTLA-4 transduced HeLa or Jurkat cells were transfected with Rab-GFP constructs (empty vector - EV; wild type - WT; dominant negative - DN; or constitutively active - CA) for 24hrs, and then stained with anti-CTLA-4 PE at 37°C for 1hr. Cells were then incubated at 37°C for up to 4hrs, before being fixed, and analysed by flow cytometry. **A,C:** Flow cytometry histogram of HeLa (A) or Jurkat (C) cells showing GFP+ gate used for analysis. **B,D:** Graphs showing CTLA-4 staining in GFP+ gated HeLa (B) or Jurkat (D) cells, relative to 0hrs. Data shown as mean ± SD, n ≥ 3. Differences between conditions were determined by two-way ANOVA and Dunnett’s multiple comparisons test; *p<0.05, **p<0.01, **** p<0.001, ns - non-significant.

CTLA-4 is known to have a short half-life and to degrade in lysosomes (27). To determine the influence of Rab GTPases on CTLA-4 degradation we pulsed cells with anti-CTLA-4-PE at 37°C for 1 hour to label CTLA-4 and then washed and incubated at 37°C to allow the antibody-bound CTLA-4 to traffic around the cell. The cells were then fixed and permeabilised and analysed by flow cytometry for residual CTLA-4 staining, again gating on defined ‘GFP+’ populations to control for the expression level of the Rabs (Fig. 4A-D). This revealed that CTLA-4 degradation as measured by loss of CTLA-4 staining was reduced in cells overexpressing Rab5 DN, Rab5 CA and Rab7 DN. The sensitivity of this assay to the lysosomal inhibitor BafA1 confirmed that the loss of CTLA-4 staining was due to degradation, and not just PE signal quenching (Fig. S3). The Rab7 DN data support the hypothesis that CTLA-4 traffics through late endosomes as part of its degradation trafficking pathway. As seen in (Fig. 3), Rab5 DN inhibits endocytosis and increases cell surface expression, thereby inhibiting the first steps towards degradation. Somewhat counter-intuitively Rab5 CA was the most potent in preventing CTLA-4 degradation. However Rab5+ early endosomes mature into Rab7+ late endosomes in a process that requires the inactivation of Rab5. As Rab5 CA is never inactivated, early endosomes are able to fuse with each other, but may not mature into late endosomes, thereby preventing CTLA-4 degradation. Interestingly, none of the Rab11 constructs affected CTLA-4 degradation, suggesting Rab11 is not involved in this aspect of CTLA-4 trafficking. The impact of manipulating Rab GTPases was again similar in both cell types providing independent confirmation for the above observations although the effects in Jurkat cells were somewhat more pronounced (Fig. 4B vs D).

### Rab11 regulates CTLA-4 recycling

**Figure 5:**
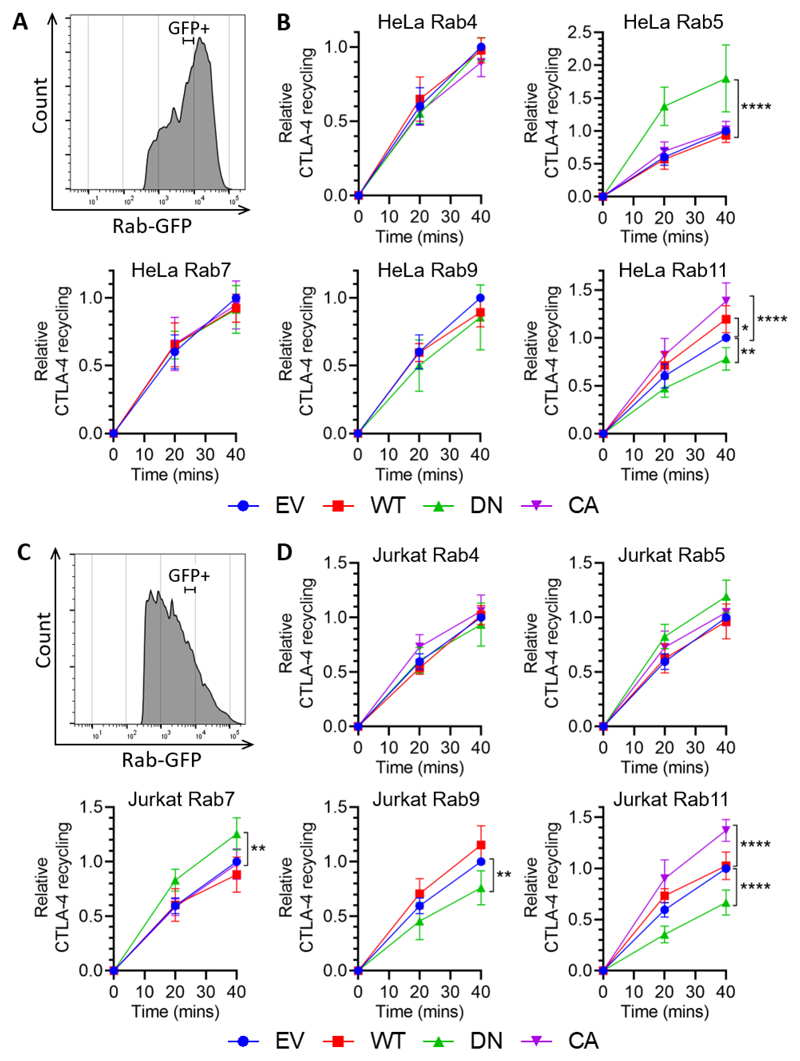
CTLA-4 recycling is regulated by Rab11 CTLA-4 transduced HeLa or Jurkat cells were transfected with Rab-GFP constructs (empty vector - EV; wild type - WT; dominant negative - DN; or constitutively active - CA) for 24hrs, and then stained with human anti-CTLA-4 at 37°C for 1hr, and detected by anti-human IgG-AlexaFluor647 at 37°C for the times shown. **A,C:** Flow cytometry histogram of HeLa (A) or Jurkat (C) cells showing Rab-GFP+ gate used for analysis. **B,D:** Graphs showing recycling CTLA-4 staining in GFP+ gated HeLa (B) or Jurkat (D) cells, relative to EV. Data shown as mean ± SD, n ≥ 3. Differences between conditions were determined by two-way ANOVA and Dunnett’s multiple comparisons test; *p<0.05, **p<0.01, **** p<0.001.

Recycling is believed to be important for CTLA-4’s regulatory function, so next we assessed the effects of Rab overexpression on CTLA-4 recycling. To assay the recycling, cells were pulsed with an unlabelled human anti-CTLA-4 for 1hr at 37°C to label any CTLA-4 before it is internalised. Cells were then stained with anti-human IgG-AlexaFluor 647 on ice to detect residual surface CTLA-4 providing a baseline, and then labelling was continued at 37°C to detect any antibody-bound CTLA-4 as it recycles to the cell surface. Accordingly, staining increased with time as CTLA-4 was recycled. In both HeLa and Jurkat cells, Rab11 DN overexpression decreased recycling while Rab11 CA increased recycling (Fig. 5). This provides direct support for the idea that CTLA-4 is recycled via a Rab11 regulated pathway in line with the role of Rab11 in recycling endosomal sorting of other receptors. In addition there were indications in Jurkat cells that Rab7 DN increased and Rab9 DN decreased CTLA-4 recycling (Fig. 5D). Given the impact of Rab7 on CTLA-4 degradation, this suggests that inhibiting CTLA-4 traffic into late endosomes and towards the degradation pathway effectively increases its entry to the recycling pathway. The Rab9 data suggest the possibility that CTLA-4 may be able to recycle from late endosomes to the TGN via the Rab9 regulated pathway (28). Indeed others have reported a role for Rab8 in transport of CTLA-4 from the TGN (29). In HeLa cells Rab5 DN increased CTLA-4 recycling particularly at early time points, which may indicate that blocking traffic from early endosomes may force CTLA-4 to enter a rapid recycling route and so increases early recycling. A similar but non-significant trend was observed in Jurkat cells. Taken together these data show considerable overlap of general principles between cell types as well as revealing subtle differences in the efficiency and sensitivity of CTLA-4 recycling depending on cell type.

### Targeting of LRBA reduces CTLA-4 expression and recycling.

**Figure 6:**
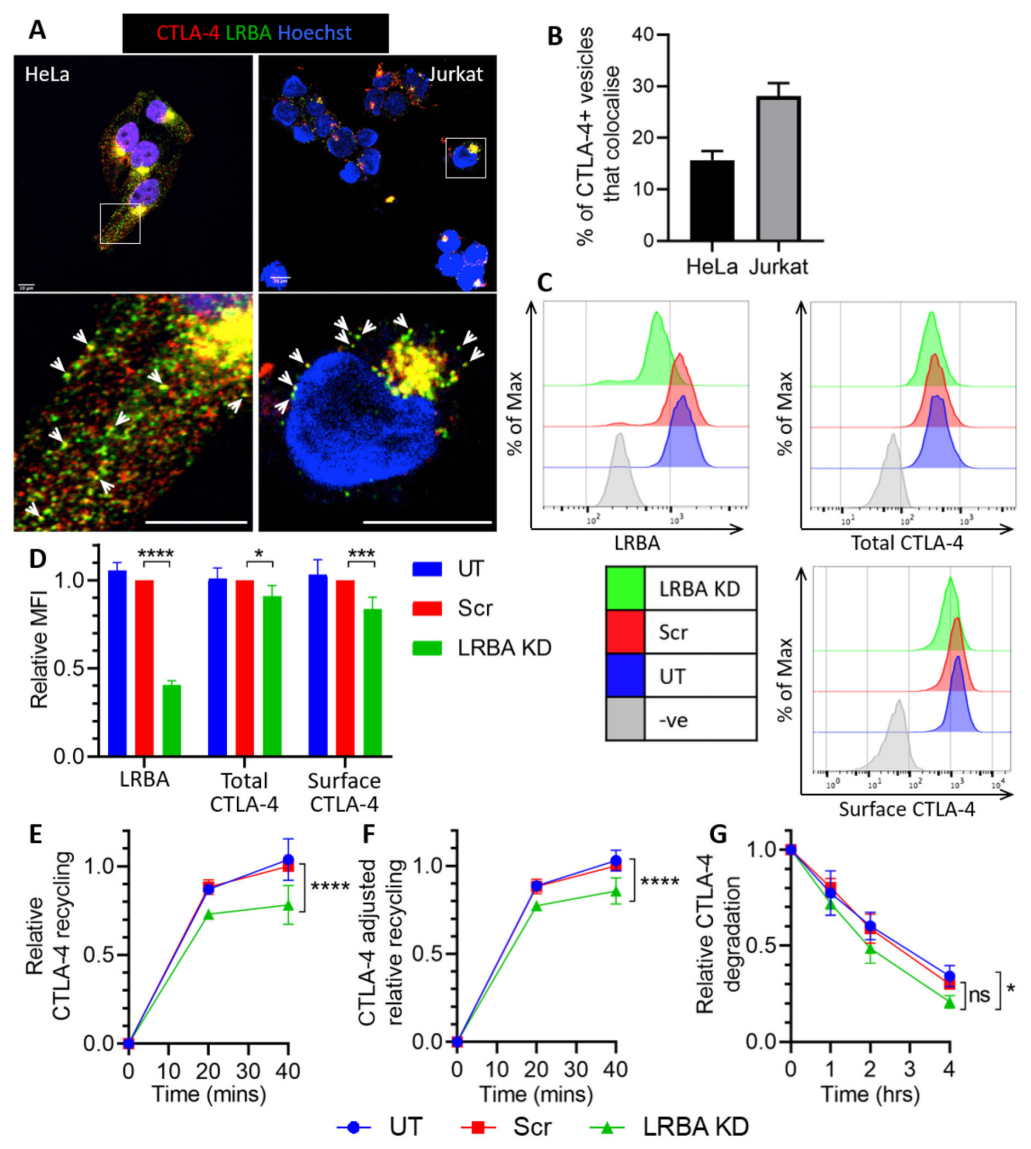
LRBA siRNA KD has a limited effect on CTLA-4 trafficking in HeLa cells **A**: CTLA-4 transduced HeLa or Jurkat cells were fixed, permeabilised and stained with anti-CTLA-4 (red) and anti-LRBA (green) and analysed by confocal microscopy. Scale bars = 10μm. **B**: Graphs showing quantification of the colocalisation of CTLA-4 vesicles with LRBA vesicles from (A). Data shown as mean ± SEM, n=3 independent experiments. **C-G:** CTLA-4+ HeLa cells were transfected with siRNA targeting LRBA or a scramble control (Scr), or untransfected (UT). **C**: Flow cytometry showing the expression of LRBA and CTLA-4 in targeted cells, quantified in **D.** Data shown as mean ± SD, n ≥ 3. **E**: siRNA transfected HeLa cells were stained for recycling CTLA-4 (as described in Fig 5). Graph shows recycling CTLA-4 staining relative to Scr 40’. **F**: Graph shows relative recycling CTLA-4 from (E) adjusted for the differences in total CTLA-4 staining level from (D). **G**: siRNA transfected HeLa cells were treated with 50μg/ml CHX for up to 4hrs and then stained for total CTLA-4 expression. Graph shows CTLA-4 staining relative to 0hrs. In (E,F&G), data shown as mean ± SD, n ≥ 3. In (D) differences between conditions and Scr control determined by one-way ANOVA and Dunnett’s multiple comparisons test, and in (E,F&G) differences between conditions determined by two-way ANOVA and Tukey’s multiple comparisons test; * p<0.05, *** p<0.005, **** p<0.001, ns - non-significant.

LRBA has recently been proposed to play a critical role in regulating CTLA-4 trafficking, but our understanding is still limited (6). We therefore investigated whether LRBA regulates CTLA-4 trafficking in our cell lines. Initially we stained CTLA-4 transduced HeLa and Jurkat cells for LRBA and assessed by confocal microscopy whether the two proteins colocalised (Fig. 6A). CTLA-4 and LRBA clearly colocalised, both in the perinuclear region as well as in more peripheral vesicles, and quantification showed that the colocalisation was higher in the Jurkat cells (28%) than HeLa cells (15%) (Fig. 6B). We next assayed the effect of LRBA knockdown on CTLA-4 expression in HeLa cells using siRNA (Fig. 6C&D). siRNA knockdown reduced LRBA expression by at least 60%. Despite this we only observed a limited (10%) reduction in total CTLA-4 and a 16% reduction in surface CTLA-4 expression.

LRBA KD significantly decreased CTLA-4 recycling over 40 minutes (-22%), even when the recycling was normalised to the total CTLA-4 expression (-14%) (Fig. 6E&F). In addition we observed a small increase in CTLA-4 degradation when compared to the untreated control (Fig. 6G). Together these data suggest that LRBA plays a role in the decision between CTLA-4 recycling and degradation, affecting total CTLA-4 expression levels, but that LRBA knockdown in HeLa cells did not generate a strong phenotype. Given that the phenotype of patients with LRBA deficiency requires a homozygous defect, it is plausible that the siRNA knockdown was insufficient to reveal the impact of LRBA.

**Figure 7:**
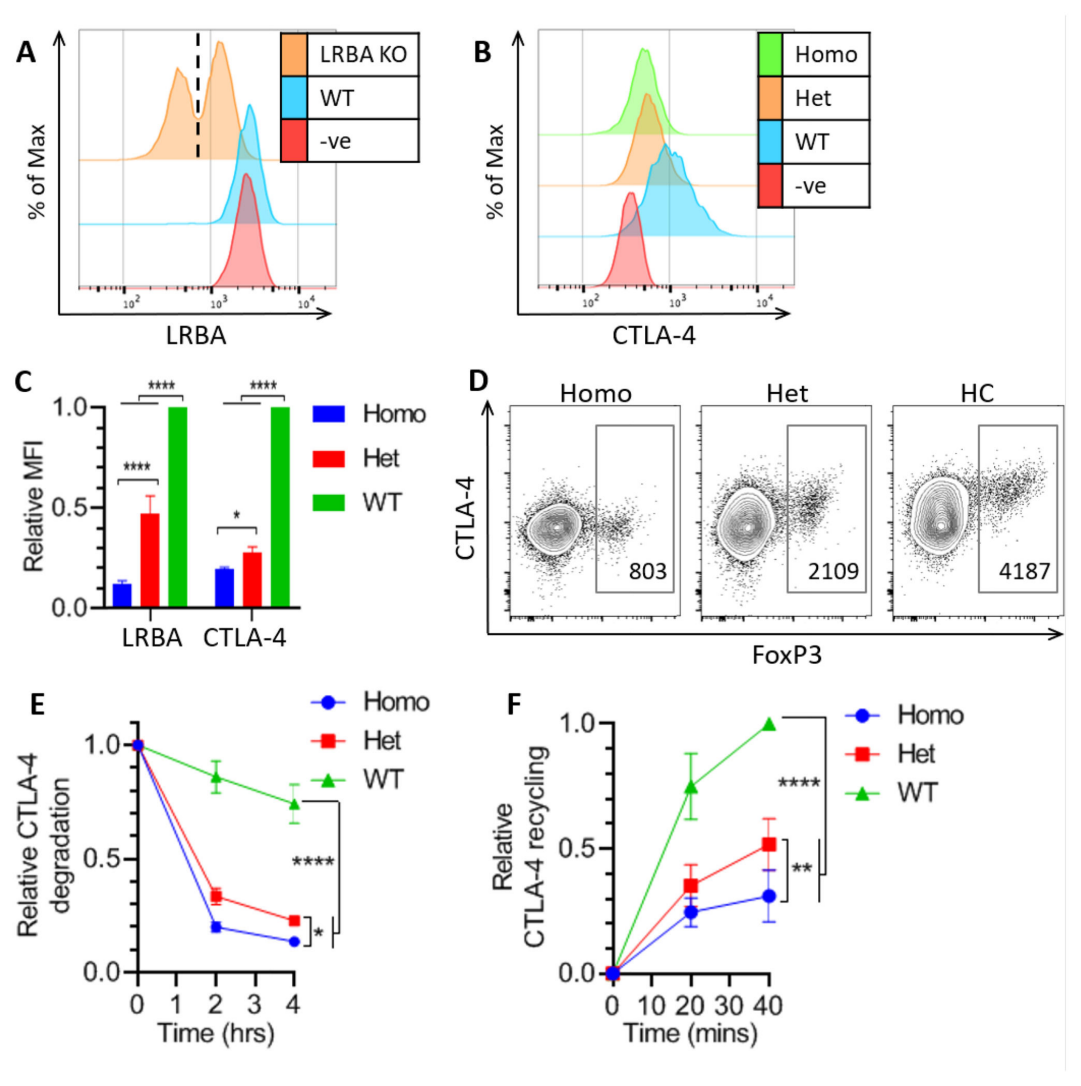
LRBA regulates CTLA-4 recycling and degradation in Jurkat cells **A-C:** CTLA-4 negative (-ve), CTLA-4 transduced (WT), or CTLA-4 transduced LRBA CRISPR KO Jurkat cells were fixed, permeabilised and stained for LRBA and total CTLA-4. **A**: Flow cytometry histogram showing LRBA expression in -ve, WT and LRBA KO Jurkat cells. Dashed line marks boundary between homozygous (homo) and heterozygous (het) populations. **B**: Histograms showing CTLA-4 expression in -ve, WT, and homo/het LRBA KO Jurkat cells. **C**: Cumulative data of the relative LRBA and CTLA-4 MFI in LRBA KO Jurkat cells. Data shown as mean ± SD, n=4. **D**: Flow cytometry plots showing FoxP3 and CTLA-4 staining in CD4+ CD45RA- T cells from individuals with homozygous or heterozygous LRBA mutation, or healthy control (HC). CTLA-4 MFI of the FoxP3+ Tregs is shown. **E**: WT and LRBA KO Jurkat cells were treated with 50μg/ml CHX for up to 4hrs, and stained for total CTLA-4. Graph shows total CTLA-4 staining relative to 0hrs. **F**: WT and LRBA KO Jurkat cells were stained for recycling CTLA-4. and plotted relative to WT 40’. Data shown as mean ± SD, n=3. In (C,E&F) differences between conditions determined by two-way ANOVA and Tukey’s multiple comparisons test; * p<0.05, ** p<0.01, **** p<0.001.

Given this, we used a CRISPR/Cas9 approach to knockout LRBA from CTLA-4 transduced Jurkat cells and then stained them for LRBA and total CTLA-4 (Fig. 7A&B). The LRBA staining showed that the CRISPR knockout was successful and produced both complete homozygous KO cells and heterozygous KO cells which retained ~50% LRBA expression (Fig. 7A). We then gated on these different populations to examine the effects of the different LRBA levels on CTLA-4 (Fig. 7B). The loss of LRBA caused a greater decrease in CTLA-4 expression in Jurkat cells compared to HeLa cells, with heterozygous cells also expressing significantly lower levels of CTLA-4 (Fig. 6D & Fig. 7C). This pattern of CTLA-4 expression was similar to our observations in Treg from patients with LRBA mutations (Fig. 7D) where we also observe an effect of heterozygosity, albeit less severe than the loss of CTLA-4 in the LRBA homozygous patient. Next we investigated the impact of loss of LRBA on CTLA-4 degradation and recycling in Jurkat cells (Fig. 7 E&F). The LRBA KO cells showed greatly increased CTLA-4 degradation and decreased recycling, with the homozygous KO cells being significantly more affected than the heterozygous KO cells in both assays. In the recycling assay, normalisation to total CTLA-4 levels still revealed decreased recycling in the absence of LRBA (Fig. 7F). These data indicate that LRBA plays a key role in the CTLA-4 recycling pathway in Jurkat cells and is either involved in the recycling pathway itself or (given the degradation phenotype) in the decision point between recycling and degradation.

### Increasing Rab11 activity fails to rescue CTLA-4 recycling in LRBA KO cells

**Figure 8:**
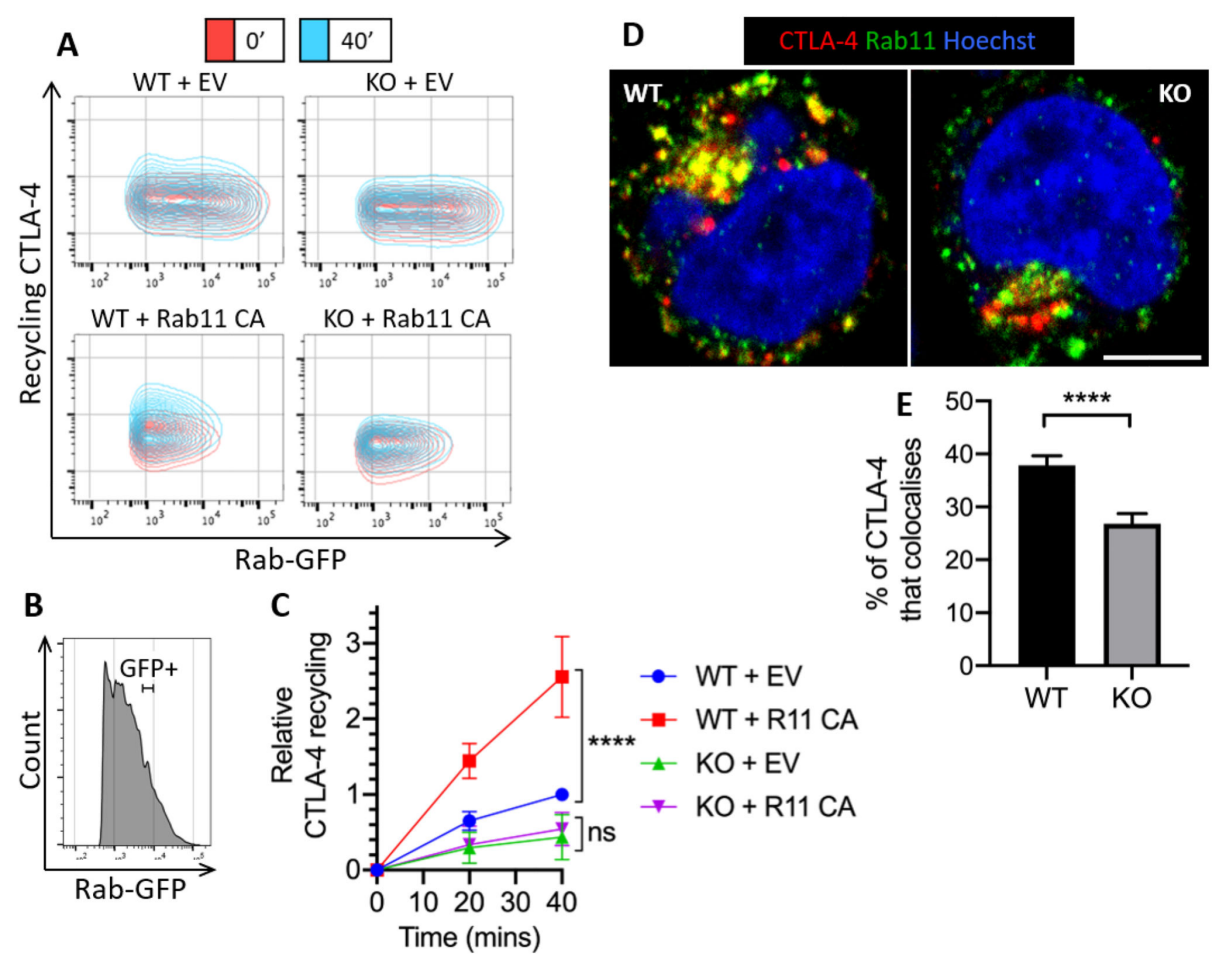
Increasing Rab11 activity fails to rescue CTLA-4 recycling in LRBA KO cells WT or LRBA KO CTLA-4 transduced Jurkat cells were transfected with Rab11-Constitutively Active (CA) GFP, or empty vector (EV), and stained for recycling CTLA-4. **A**: Flow cytometry plots showing recycling CTLA-4 staining. **B**: Flow cytometry histogram of Jurkat cells showing Rab- GFP+ gate. **C**: Graph showing cumulative data of relative CTLA-4 recycling in WT or LRBA KO cells transfected with Rab11 CA-GFP or EV, gated on Rab-GFP expression. Data shown as mean ± SD, n ≥ 3. Differences between conditions determined by two-way ANOVA and Tukey’s multiple comparisons test; **** p<0.001, ns - non-significant. **D**: WT or LRBA KO CTLA-4 transduced Jurkat cells were fixed, permeabilised and stained with anti-CTLA-4 (red) and anti-Rabll (green) and analysed by confocal microscopy. Scale bar = 5μm. **E**: Graph showing quantification of the percentage of CTLA-4 intensity in vesicles colocalising with Rab11 vesicles from (D). Data shown as mean ± SEM, n = 4 independent experiments. Difference between conditions determined by unpaired T-test; **** p<0.001.

Since overexpression of Rab11 CA increases CTLA-4 recycling, we tested whether Rab11 CA could rescue the decreased recycling seen in the LRBA KO Jurkat cells. To do this, we transiently transfected either WT or LRBA KO Jurkat cells with the Rab11 CA-GFP construct and performed a recycling assay (Fig. 8A). To quantify the recycling, we measured the recycling CTLA-4 MFI from cells within a defined ‘GFP+’ gate that was equivalent between conditions (Fig. 8B&C). Interestingly, although Rab11 CA expression caused a 2.5x increase in recycling in the WT cells after 40 minutes, the LRBA KO cells showed no such effect. This indicated that loss of LRBA was limiting for CTLA-4 recycling, and, coupled with the increased CTLA-4 degradation seen previously in LRBA KO cells (Fig. 7E), suggested that LRBA was involved in controlling CTLA-4 fate upstream of Rab11. To confirm where LRBA functions in the CTLA-4 recycling pathway, we stained either WT or LRBA KO cells for CTLA-4 and Rab11 colocalisation and imaged them by confocal microscopy (Fig. 8D). If LRBA were downstream of Rab11, we reasoned that LRBA KO cells would be expected to show increased colocalisation between CTLA-4 and Rab11, as the recycling CTLA-4 would be trapped in the Rab11+ compartment. In contrast, if LRBA were upstream of Rab11, we would predict that the colocalisation between CTLA-4 and Rab11 would be reduced, as CTLA-4 would be unable to reach the Rab11+ compartment. Quantification of colocalization (Fig. 8E) showed that Rab11-CTLA-4 colocalisation was significantly lower in the LRBA KO cells, supporting the concept that LRBA functions upstream of Rab11.

## 5 Discussion

**Figure 9:**
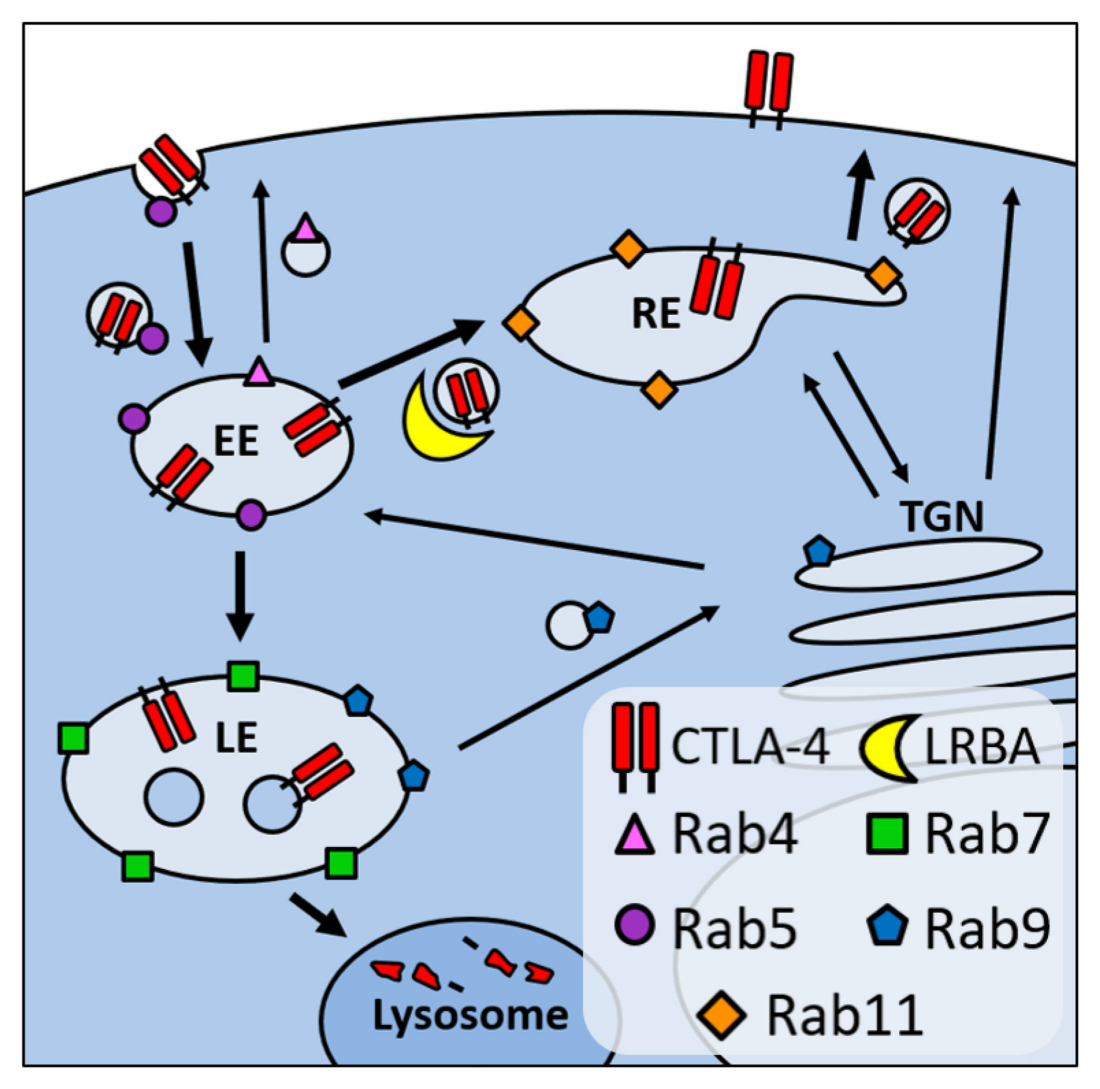
Model of CTLA-4 trafficking Diagram shows the proposed relationship between CTLA-4, LRBA and different Rab GTPases. EE - early endosome, LE - late endosome, RE - recycling endosome, TGN - trans Golgi network. Bold arrows mark pathways used by CTLA-4.

CTLA-4 forms a vital checkpoint in the regulation of T cell activation (30,31), inhibiting CD28 costimulation by capturing and internalising its shared ligands via transendocytosis (15). The intracellular trafficking of CTLA-4 is therefore believed to be important for its function but this is not well understood. Here we set out to study the pathways utilised by CTLA-4 for its recycling and degradation and the cellular proteins which regulate this trafficking. A schematic model of our findings is shown in Fig. 9. We investigated CTLA-4 trafficking in Jurkat T cells and HeLa cells and observed similar results in both cell types, indicating that the regulatory mechanisms involved in CTLA-4 trafficking are broadly conserved.

We found that CTLA-4 colocalises with a panel of endosomal Rab GTPases as it distributes across multiple cellular compartments, and that overexpression of GFP-tagged Rab proteins alters CTLA-4 trafficking. Rab5 has been shown to regulate endocytosis of plasma membrane proteins and to promote homotypic fusion of early endosomes (17,32). In this study we observed that inhibition of Rab5 activity increased CTLA-4 surface expression and reduced its degradation which suggests that CTLA-4 is endocytosed and trafficked via a similar pathway. Rab7 is known to be recruited to early endosomes and regulate maturation into late endosomes, where it has been shown to control protein transport from these late endosomes to lysosomes (21,33). Our observation that inhibition of Rab7 activity reduced CTLA-4 degradation indicates that CTLA-4 degrades via the Rab7-regulated late endosome-lysosome pathway in a manner similar to that used for EGFR degradation (34). This is consistent with previous data showing that CTLA-4 degradation is blocked by lysosomal inhibitors (12,22).

Rab11 is known to regulate recycling via the pericentriolar recycling endosome (19). We found that increasing Rab11 activity increases both CTLA-4 recycling and surface expression, while inhibiting its activity has the opposite effect. This suggests that CTLA-4 recycles via the classical recycling pathway, as used by receptors such as transferrin receptor and recycling EGFR, and the Rab11- regulated step is rate limiting in CTLA-4 recycling (26,35). As such Rab11 influences the amount of CTLA-4 at the plasma membrane and so this specific step may be of interest in potential therapies that hope to more subtly regulate CTLA-4 function, to treat autoimmune conditions or cancer.

LRBA has recently been shown to be critical for CTLA-4 biology, and patients with homozygous LRBA mutations have reduced CTLA-4 expression (6,36). In keeping with the data of Lo et.al., we show that loss of LRBA decreases CTLA-4 recycling and increases its degradation. Interestingly, homozygous LRBA mutations appear to be completely penetrant in causing disease, whereas patient relatives who have heterozygous mutations are usually asymptomatic (37). This suggests that in humans, a single functional LRBA allele is able to maintain sufficient CTLA-4 function despite CTLA-4 expression being reduced. Interestingly, the importance of LRBA in regulating CTLA-4 appears to vary in different species and cell types since LRBA KO mice showed limited pathology (38,39). Accordingly, we found that loss of LRBA in Jurkat cells had a much greater effect on CTLA-4 than in HeLa cells. One possibility is that the requirement for CTLA-4 recycling may be more demanding in humans than in mice or alternatively that LRBA knockout mice continue to recycle sufficient CTLA-4 via other means. Regarding the importance of CTLA-4 recycling, we have previously observed that in patients with LRBA deficiency the low levels of CTLA-4 are readily mitigated by T cell stimulation resulting in strong upregulation of CTLA-4 expression (36). The fact that this does not correct CTLA-4 function *in vivo* may indicate that it is the recycling of CTLA-4, not just its expression level, which is critical for effective function. Finally, we also demonstrate that Rab11 CA overexpression was unable to increase recycling in LRBA KO cells which suggests LRBA acts upstream of Rab11 in the CTLA-4 recycling pathway. This is supported by the observation that LRBA deficiency reduced the presence of CTLA-4 in Rab11 compartments. The fact that LRBA appears to work upstream of Rab11 suggests that the defect in LRBA deficiency is one of committing CTLA-4 to the Rab11 recycling pathway and therefore causing CTLA-4 to default to a degradative fate.

Taken together the data presented here provide an enhanced view of the critical steps in CTLA-4 trafficking and suggest new possibilities for manipulating CTLA-4 expression and function.

## Supplementary Material

S1-S3

## Data Availability

The data that support the findings of this study are available from the corresponding author upon reasonable request.

## Abbreviations

APC: Antigen-presenting cell
BafAl: Bafilomycin A1
CA: Constitutively active
CTLA-4: Cytotoxic T lymphocyte associated antigen-4
DN: Dominant negative
KD: Knockdown
KO: Knockout
LRBA: LPS-responsive beige-like anchor protein
TGN: Trans-Golgi network
Treg: Regulatory T cell
WT: Wild type
