# Supplementary material for: Regulation of CTLA-4 recycling by LRBA and Rab11
Source: Immunology. Author manuscript; Available in PMC 2021 Sep 1. (PMC8358724; doi:10.1111/imm.13343)
Supplement: S1-S3 [file EMS126082-supplement-S1_S3.pdf]

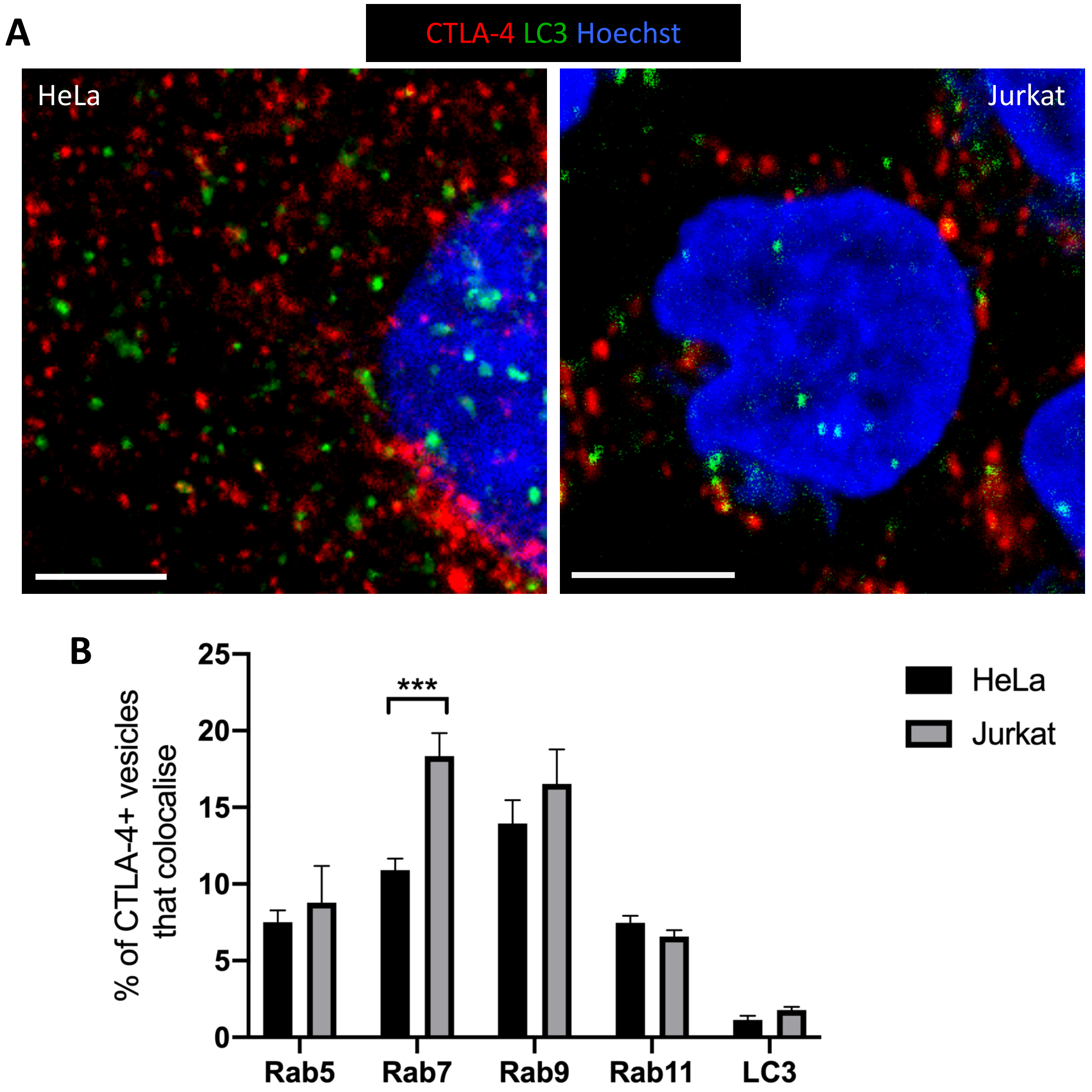

Figure S1: CTLA-4 does not colocalise with autophagy marker protein LC3

A: CTLA-4 transduced HeLa or Jurkat cells were fixed, permeabilised and stained with human anti-CTLA-4 and rabbit anti-LC3 Abs followed by goat anti-human IgG-AlexaFluor546, donkey anti-rabbit IgG-AlexaFluor488, Hoechst and CTV. Cells were analysed by confocal microscopy. Scale bars = 5µm. **B:** Graph showing quantification of the colocalisation of CTLA-4 vesicles with LC3 vesicles in (A) or Rab vesicles (from Fig 2). LC3 Data shown as mean ± SEM, n ≥ 8 fields of view.

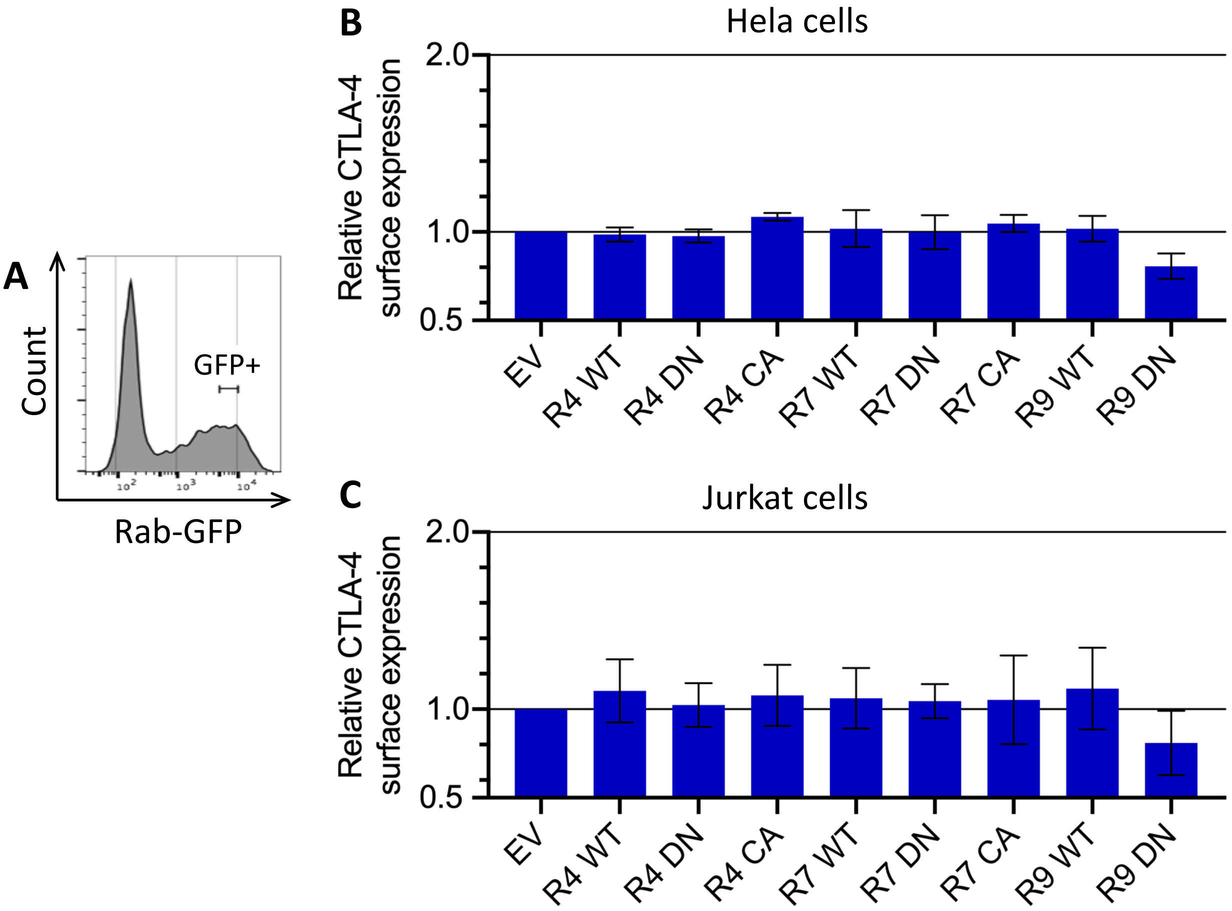

Figure S2: CTLA-4 surface expression is not regulated by Rab4, Rab7 or Rab9

CTLA-4 transduced HeLa or Jurkat cells were transfected with Rab-GFP constructs (empty vector: EV, wild type: WT, dominant negative: DN, or constitutively active: CA) for 24hrs, and then stained for surface CTLA-4 expression. **A:** Flow cytometry plot showing the GFP+ gate used. **B,C:** Graphs showing surface CTLA-4 expression relative to empty vector control in GFP+ gated HeLa (B) or Jurkat (C) cells. Data shown as mean ± SD, B: n ≥ 3, C: n ≥ 4. Differences between conditions and EV determined by one-way ANOVA and Dunnett's multiple comparisons test.

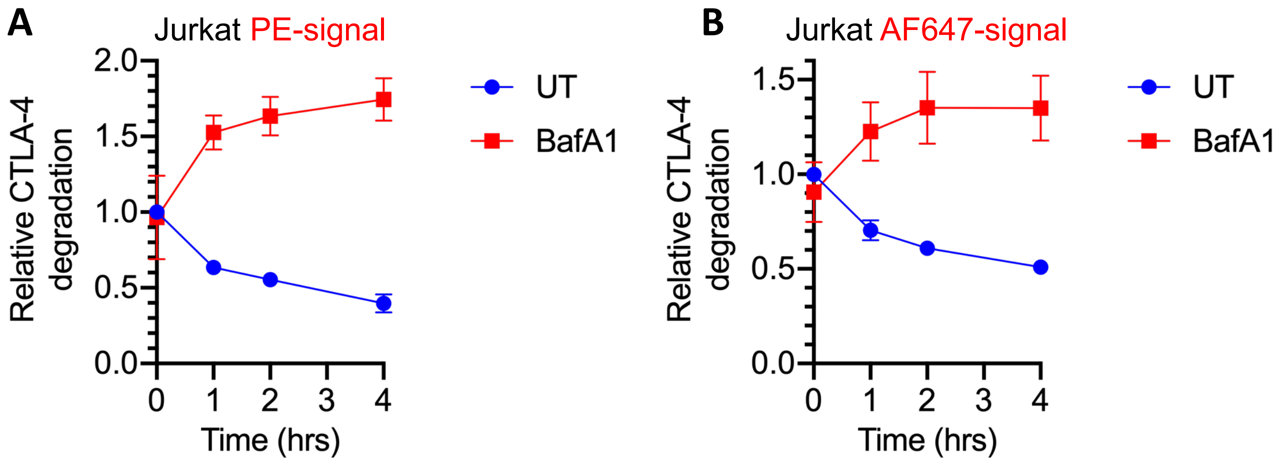

Figure S3: CTLA-4 degradation is sensitive to lysosomal inhibition

CTLA-4 transduced Jurkat cells were stained with mouse anti-CTLA-4 PE at 37°C for 1hr. Cells were then washed and incubated at 37°C for up to 4hrs, either untreated (UT) or with 10nM BafA1, before being fixed, stained with anti-mouse IgG-AlexaFluor647, and analysed by flow cytometry. **A,B:** Graphs showing direct anti-CTLA-4-PE staining (A) or goat anti-mouse IgG-AlexaFluor647 secondary antibody detection of the primary PE antibody (B) in Jurkat cells. Data shown are MFI relative to untreated cells at 0hrs, as mean ± SD, n = 3 independent experiments.
